# The effect of exercise in a fasted state on plasma low‐density lipoprotein cholesterol concentrations in males and females

**DOI:** 10.1113/EP091005

**Published:** 2023-02-21

**Authors:** Louise Bradshaw, Francoise Koumanov, Sarah Berry, James A. Betts, Javier Gonzalez

**Affiliations:** ^1^ Centre for Nutrition, Exercise & Metabolism, Department for Health University of Bath Bath UK; ^2^ Department of Nutritional Sciences, School of Life Course Sciences, Faculty of Life Sciences and Medicine King's College London London UK

**Keywords:** lipid metabolism, lipids, physical activity

## Abstract

Cardiovascular disease (CVD) is the leading cause of death worldwide. Physical activity interventions improve almost all modifiable CVD risk factors, but the effect of physical activity on low density lipoprotein cholesterol (LDL‐C) is uncertain. This may be due to lack of research on the feeding status in which the physical activity is performed. The aim of this study is to investigate the effect of fasted versus fed exercise on LDL‐C concentrations in males and females. One hundred healthy participants, equal males and females, aged between 25 and 60 years will be recruited and will undergo a home‐based 12‐week exercise intervention. After baseline testing, participants will be randomized to a fasted exercise (exercise after an 8‐h fast) or fed exercise (exercise 90–180 min after ingestion of 1 g kg^−1^ CHO) group and will perform 50 min of moderate intensity exercise (e.g., 95% heart rate of lactate threshold 1) three times a week either before or after a high carbohydrate (1 g kg^−1^) meal. Participants will visit the laboratory again at week 4 and week 12 and measurements will be taken for body composition, resting blood pressure, fasting blood glucose, lipid profiles and systemic inflammation, lactate threshold, and 14‐day blood glucose control.

## 1 INTRODUCTION

Cardiovascular disease (CVD) is the leading cause of death worldwide and accounts for 27% of all deaths in the UK (British Heart Foundation, 2020). It is well established that hyperlipidaemia is associated with an increased risk of CVD, with elevated non‐high density lipoprotein cholesterol (non‐HDL‐C) having the highest attributable risk for CVD in high‐income countries relative to other modifiable risk factors (Yusuf et al., 2020). Moreover, it has been consistently shown that lowering low density lipoprotein cholesterol (LDL‐C) concentrations proportionately lowers CVD events across the entire range of LDL‐C concentrations (Ference et al., 2017).

Physical activity is an attractive strategy to reduce population CVD risk, with clear evidence that becoming more active improves almost all CVD risk factors, such as insulin sensitivity, waist circumference, aerobic fitness, blood pressure and HDL‐C concentrations, and shows an inverse linear relationship with all‐cause and CVD‐related mortality (Blair et al., 1995; Dela et al., 1992; Fagard, 2001; Kokkinos et al., 1995; McDonough et al., 1970). It is, therefore, a notable exception that LDL‐C concentrations exhibit a far less consistent response to changes in physical activity than all other CVD risk factors (Hespanhol Junior et al., 2015; Wewege et al., 2018).

The feeding status in which physical activity is performed has a profound effect on metabolism, which may affect the ability of physical activity to lower LDL‐C concentrations. Carbohydrate intake before and/or during exercise supresses fat oxidation during exercise partly via insulin‐mediated inhibition of net adipose and intramuscular lipolysis, increased re‐esterification rates, and thus reductions in non‐esterified fatty acid (NEFA) availability (Coyle et al., 2001; Enevoldsen et al., 2004; Frayn et al., 2012). Since high lipid turnover in adipocytes is linked to a lower atherogenic lipoprotein profile (Frayn et al., 2012), the reduction in fat oxidation, and thus lipid turnover, during exercise following meal ingestion may explain the inconsistent findings on the effect of exercise on LDL‐C concentrations.

LDL‐C is produced as very low density lipoprotein (VLDL) particles become lipid depleted following hydrolysis by lipoprotein lipase (LPL; Jackson et al., 1976), leading to formation of small dense LDL‐C particles that have a low affinity for LDL receptors (Packard et al., 2000). An acute bout of fasted exercise results in changes in VLDL‐triacylglycerol (TAG) flux compared to fed exercise (Sondergaard et al., 2011), suggesting that reduction in VLDL‐TAG concentrations results from reduced hepatic production or increased skeletal uptake by lipoprotein lipase. Furthermore, a reduction of intramuscular triglyceride (IMTG) as a result of an acute bout of fasted exercise (Edinburgh et al., 2020) could increase LPL activity to replenish IMTG stores (Hardman, 1998). An acute bout of fasted exercise that reduces VLDL‐TAG performed regularly with exercise training may lower VLDL exposure over time, by either reduced hepatic VLDL production or increased LPL activity, thus reducing LDL‐C concentrations.

Biological sex is an important variable in metabolic responses, and thus responses to exercise and feeding should be considered within the context of potential sex differences. It has previously been shown that there are defined differences in lipoprotein metabolism between human males and females (Palmisano et al., 2018) due to endogenous oestrogens, which may influence disease risk of diabetes and CVD. During exercise in a fasted‐state, females can oxidise more fatty acids (FA) per kg of fat‐free mass compared to males exercising at the same relative intensity (Chrzanowski‐Smith et al., 2021). This higher utilisation of FA during exercise in females is due to upregulation of metabolic pathways related to NEFA and TAG uptake, storage and degradation in skeletal muscle (Horton et al., 2002; Lundsgaard & Kiens, 2014). During exercise in the fed‐state, however, it remains unknown whether males and females differ in the suppression of fat oxidation. Given the potential of fat oxidation to reduce atherogenic lipoprotein profiles, the greater fat oxidation in females during fasted exercise could enhance the effect of exercise in a fasted‐state on LDL‐C concentrations.

Our preliminary research (*n* = 30) indicated that exercise performed in the morning before breakfast significantly increased whole‐body lipid oxidation during an acute bout of exercise, and this effect persisted throughout 6 weeks of exercise training sessions (Edinburgh et al., 2020). The group exercising before breakfast (*n* = 9) exhibited a significant reduction in LDL‐C concentrations of 0.41 mmol l^−1^ at the end of the study compared to baseline, providing a similar reduction in LDL‐C concentrations to non‐statin drug therapies (Silverman et al., 2016). In contrast, the group who performed exercise after breakfast saw no change in their LDL‐C concentrations, suggesting feeding status at time of exercise may unlock the ability of exercise to improve LDL‐C concentrations. However, no study to date has directly assessed whether meal timing in relation to exercise alters LDL‐C concentrations following training. Furthermore, it is yet to be assessed if these changes occur in a home‐based training programme over a longer period of time and if biological sex influences the response of meal timing. Therefore, the primary aim of this randomized, parallel study is to assess the effect of physical activity performed before or after a meal on plasma LDL‐C concentrations in males and females. The secondary aim is to assess the effect of physical activity performed before or after a meal on other CVD risk factors. In our preliminary research a difference in response to an oral glucose tolerance test was also observed between the two groups but glucose control over the study period was not assessed. In addition, given that the gut microbiome has been shown to influence exercise adaptations (Mailing et al., 2019), we will explore changes in microbiome species enrichment.

## 2 METHODS

### 2.1 Study design

**FIGURE 1:**
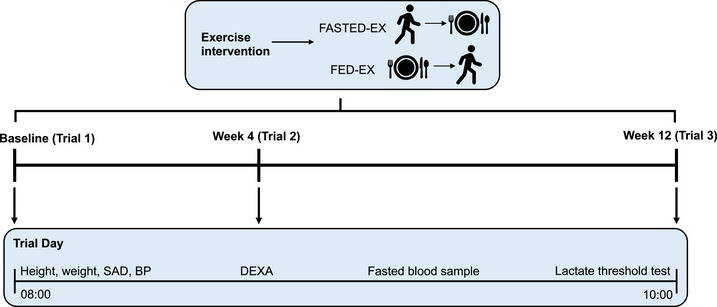
Schematic representation of study design. BP, blood pressure; DEXA, dual‐energy X‐ray absorptiometry; SAD, sagittal abdominal diameter.

This study was approved by Bristol Research and Ethics Committee (22/SW/0061) and registered on ClinicalTrials.gov (ID NCT05279014). The study design will be a randomized, parallel study of 100 inactive males (*n* = 50) and females (*n* = 50) conducted over a 2‐year period (Figure 1). The intervention will be a 12‐week exercise programme in which participants will be randomized to exercise either in a fasted state (FASTED‐EX) or in a fed state (FED‐EX). Participants in the FASTED‐EX group will perform exercise sessions at least 8 h after the last meal to ensure they are in a fasted state (Ruge et al., 2009) and eat a carbohydrate‐based meal after exercise. Participants in the FED‐EX group will perform the exercise sessions 90–180 min after the carbohydrate‐based meal. The sessions will be completed in either the morning or the evening and participants will be stratified for randomisation based on their preference of exercise timing. The participants will attend the physiology laboratory at the University of Bath on three occasions, at baseline, week 4 and upon completion of the intervention (i.e., week 12). The study will be conducted in accordance with the *Declaration of Helsinki* (Figure 1).

### 2.2 Recruitment

Participants will be recruited by advertisement on the University of Bath website and via posters and adverts throughout the campus (e.g., digital advertisement platforms, university notice boards). Posters will also be placed throughout the local community such as in libraries, community centres and health centres (e.g., GP practices, pharmacies and dentists). The study will also be advertised via email bulletin to the University of Bath students and staff and via social media (e.g., Twitter, Instagram and Facebook). Individuals interested in participating in the study will be asked to contact the principal investigator and will be provided with a participant information sheet. Potential participants will be invited to the university for initial screening and will provide written informed consent prior to commencing study procedures.

### 2.3 Randomization

Participants will be randomly allocated into two groups (FASTED‐EX or FED‐EX) using online randomisation software (www.randomization.com) with a 1:1 allocation ratio. Stratification factors will be high (30–40 kg m^−2^) or low (20–29.9 kg m^−2^) body mass index and preference of morning or afternoon exercise.

### 2.4 Participants and eligibility criteria

Participants recruited will be between the age of 25 and 60 years old, have a body mass index between 20 and 40 kg m^−2^ and be self‐reported physically inactive (exercise for less than 150 min per week). Female participants will be self‐reported premenopausal. The body mass index was based on representing the general population, whilst a physically inactive criterion was chosen to reflect individuals who are at somewhat of an increased risk of CVD. Individuals who self‐report a diagnosis of diabetes or CVD, take any medication that could pose undue personal risk or introduce bias into the study such as statins for lipid disorders, or have cardiovascular contraindications to exercise testing will be excluded. Individuals with weight instability (>3 kg change in body mass over last 6 months) or females who are pregnant or lactating will be excluded.

### 2.5 Outcome measures

An intention‐to‐treat analysis will be reported for all outcome measures. The primary outcome measure will be change in fasting plasma LDL‐C concentrations at week 12. Secondary outcomes include change in fasting LDL‐C concentrations at week 4, and change in fasting plasma HDL‐C concentrations, fasting plasma VLDL‐rich triglyceride concentrations, fasting plasma total cholesterol concentrations, fasting plasma apolipoprotein B concentrations, apolipoprotein B/apoliporotein A ratio, fasting plasma triglyceride concentrations, plasma C‐reactive protein concentrations, fasting plasma glucose concentrations, fasting plasma insulin concentrations and fasting plasma NEFA concentrations at week 4 and 12. Tertiary outcomes measures include change in body mass, waist to hip ratio, fat mass, fat‐free mass, sagittal abdominal diameter, and systolic and diastolic blood pressure at week 4 and 12, as well as change in gut microbiome species enrichment at week 12 and 14‐day blood glucose control at week 11 and 12. Secondary outcomes are subject to funding.

### 2.6 Laboratory assessments

Laboratory assessments will remain the same during all three trial days. Participants will arrive at the laboratory after an overnight fast and 24 h after any strenuous exercise and 48 h after exercise for the last visit. Participants will complete a food diary for 24 h prior to attending the laboratory and replicate this for the subsequent visits. The initial laboratory visit for eumenorrhoeic females will be performed at any stage during the menstrual cycle and due to the study protocol, follow‐up visits will be performed during the same stage, presuming a 28‐day cycle. During laboratory visits, blood pressure and body composition will be measured, followed by a blood sample and  submaximal exercise test.

#### 2.6.1 Body composition

Height will be measured using a stadiometer (Seca Ltd, Birmingham, UK) with participants barefoot in the Frankfurt plane. Body mass will be measured using digital scales (Tanita, Amsterdam, The Netherlands) with participants barefoot wearing light clothing. Waist and hip circumference will be measured to the nearest 0.1 cm using a non‐elasticated anthropometric tape (Seca, Hamburg, Germany). Waist circumference will be measured at the mid‐way point between the 12th rib and iliac crest. Hip circumference will be measured at the widest point of the buttocks. Sagittal abdominal diameter will be measured at end‐tidal volume with participants lying supine with their hips and knees flexed at 45° using an abdominal calliper (Holtain Ltd, Crymych, UK). A DEXA scan (Hologic, Marlborough, MA, USA) will be performed to determine fat mass and fat free mass. Participants will be positioned in the centre of the table with feet evenly spaced apart and the arms prone.

#### 2.6.2 Blood pressure

Blood pressure will be measured by a digital blood pressure monitor (Panasonic, Hikone, Japan). Participants will be lying supine and the measurements taken from the right arm. Three measurements 1 min apart will be obtained, and a mean taken.

#### 2.6.3 Blood analysis

Blood samples will be obtained by venepuncture from an antecubital vein and 10 ml of blood will be taken. The sample will be placed into two EDTA tubes. The samples will be centrifuged at 4000 *g* for 10 min at 4°C. The plasma will be frozen in aliquots on dry ice prior to being frozen at −80°C until further analysis.

Plasma LDL‐C, HDL‐C, total cholesterol, triglyceride, ApoB, glucose and NEFA concentrations will be analysed via spectrophotometry using a Daytona Rx (Randox, Crumlin, County Antrim, UK). Insulin and CRP concentrations will be analysed using commercially available enzyme‐linked immunosorbent assay kits. If funding is available, lipidomic analysis will be done by nuclear magnetic resonance spectroscopy to determine the full lipid profile, which will include glycoprotein acetyls.

#### 2.6.4 Blood glucose control

A continual glucose monitor (Freestyle Libre Pro iQ, Abbott Diabetes Care, Maidenhead, UK) will be fitted to the posterior aspect of the upper arm during baseline testing and worn for 14 days. A second monitor will be fitted 14 days prior to the final laboratory visit and will be removed on arrival at the laboratory. Data from the glucose monitors at week 1, week 2, week 11 and week 12 will be used to determine 14‐day coefficient of variation for blood glucose concentration as the primary analysis, as well as 14‐day mean and standard deviation of blood glucose concentration, and 14‐day mean amplitude of glycaemic excursions for blood glucose (Battelino et al., 2019).

#### 2.6.5 Microbiome

Participants will be provided with stool sample kits and asked to provide samples prior to baseline testing and end point testing. The samples will be stored and sent for analysis of gut microbiome species enrichment if funding allows. Analysis will be controlled for self‐reported antibiotic use.

#### 2.6.6 Exercise testing

Participants will be fitted with a digital heart rate monitor (Polar Electro Oy, Kempele, Finland) and perform a submaximal treadmill exercise test to determine lactate threshold. A baseline blood lactate concentration will be measured by finger prick blood analysis using Lactate Pro. The test will commence at 2.5 km h^−1^ at a gradient of 0% and consist of 6 × 4‐min incremental stages. During the first three stages the speed will be increased by 1 km h^−1^ up to 4.5 km h^−1^. The speed will then remain the same and the gradient will be increased by 4% for the remaining three stages until completion. Blood lactate concentrations will be taken during the last minute of each stage. Heart rate and rating of perceived exertion (Borg, 1970) will also be recorded during the last minute of each stage. LT1 will be determined at the point where blood lactate concentration rises by 0.5 mmol l^−1^ above resting concentrations.

#### 2.6.7 Exercise intervention

Following baseline testing, participants will undertake a 12‐week home based exercise intervention. Participants will perform 50 min of moderate intensity exercise, 3 days per week, as per current UK physical activity guidelines (Davies et al., 2019). The participant will be able to decide where to complete the exercise sessions. The exercise will be at a low‐to‐moderate intensity (e.g., 95% heart rate of LT1) to ensure greatest fat oxidation in the fasted group (Achten & Jeukendrup, 2004) and will likely represent a brisk walk for most participants. The exercise can be done either outside or on an indoor treadmill. The exercise sessions can be completed on any day of the week but participants will be asked to take at least one rest day between sessions. Participants will be asked to perform at least two out of three of the weekly sessions at the time of day stated during randomisation. The exercise intensity will be prescribed based on heart rate and will equate to 95% of LT1 heart rate. Participants will be provided with a Wahoo activity monitor (Wahoo Fitness LLC, Atlanta, Georgia) and will be asked to monitor their heart rate on the phone application during the exercise sessions. The FASTED‐EX group will perform the exercise after an 8 h fast and will ingest a high carbohydrate meal (1 g kg^−1^ body weight) after each session. The FED‐EX group will exercise 90–180 min after the same high carbohydrate meal. Participants will be able to choose the meal from a predetermined list with prescribed portion sizes ensuring the meal is similar to food they would habitually eat. The meal list includes, but is not limited to, a bowl of cereal and semi‐skimmed milk, toast with jam, bagel and low fat cream cheese, and beans on toast. Participants will be emailed every 2 weeks with their energy expenditure and heart rate during the exercise sessions to encourage compliance with the study protocol. If the intensity of exercise in which LT1 is achieved changes from week 1 to week 4, the prescribed heart rate of the exercise sessions will be amended to ensure the same relative intensity of exercise is undertaken throughout the remaining study period.

### 2.7 Statistical analysis

Statistical analysis will be performed on Prism 9 version 9.4.1 (GrahPad Software, San Diego, CA, USA). An intention‐to‐treat analysis will be performed on data of all participants who are randomized. Data will be assessed for normal distribution by inspection of residual plots. Difference between groups at weeks 4 and 12 will be assessed by ANCOVA adjusting for stratification factors, sex and baseline measurements for the whole sample (Bland & Altman, 2015). Additional exploratory analysis will be performed adjusting for change in body mass from baseline for the whole sample and subgroup analysis on males and females separately for all outcomes. Differences between sex at weeks 4 and 12 will be assessed by a two‐way ANCOVA. Statistical significance will be defined as *P* ≤ 0.05.

### 2.8 Sample size

Our preliminary data (Edinburgh et al., 2020) showed exercise before feeding resulted in a change in LDL‐C concentrations of −0.41 ± 0.51 mmol l^−1^ compared to −0.01 ± 0.046 mmol l^−1^ with exercise after feeding. Using this effect size of *d =* 0.82, 80 participants will provide >95% power at an α‐level of 0.05 with Student's two‐tailed independent *t*‐test. From previous research we expect a dropout rate of approximately 20%. To account for dropouts 100 participants (50 males and 50 females) will be recruited.

## AUTHOR CONTRIBUTIONS

Louise Bradshaw and Javier Gonzalez designed the study with input from all authors. Louise Bradshaw will collect and analyse the data. Louise Bradshaw led in writing this manuscript and all authors contributed to the review. All authors have read and approved the final version of this manuscript and agree to be accountable for all aspects of the work in ensuring that questions related to the accuracy or integrity of any part of the work are appropriately investigated and resolved. All persons designated as authors qualify for authorship, and all those who qualify for authorship are listed.

## CONFLICT OF INTEREST

J.G. is an investigator on research grants funded by BBSRC, MRC, British Heart Foundation, The Rank Prize Funds, The European Society for Clinical Nutrition and Metabolism (ESPEN), Lucozade Ribena Suntory, ARLA Foods Ingredients, Cosun Nutrition Center, and Clasado Biosciences; and has completed paid consultancy for PepsiCo and SVGC. J.A.B. is an investigator on research grants funded by BBSRC, MRC, British Heart Foundation, Rare Disease Foundation, EU Hydration Institute, GlaxoSmithKline, Nestlé, Lucozade Ribena Suntory, ARLA foods, Kennis Centrum Suiker and Salus Optima (L3M Technologies Ltd); has completed paid consultancy for PepsiCo, Kellogg's, SVGC and Salus Optima (L3M Technologies Ltd); is Company Director of Metabolic Solutions Ltd; receives an annual honorarium as a member of the academic advisory board for the International Olympic Committee Diploma in Sports Nutrition; and receives an annual stipend as Editor‐in Chief of *International Journal of Sport Nutrition & Exercise Metabolism*. S.B. receives funding from ZOE Ltd, Malaysian Health Ministry, and Almond Board of California; and receives consultancy and stock option from ZOE.

## FUNDING INFORMATION

There is no external funding for this study.
